# Crystal structure of (*E*)-2-hy­droxy-4′-meth­oxy­aza­stilbene

**DOI:** 10.1107/S2056989015008348

**Published:** 2015-05-07

**Authors:** Suchada Chantrapromma, Narissara Kaewmanee, Nawong Boonnak, Kan Chantrapromma, Hazem A. Ghabbour, Hoong-Kun Fun

**Affiliations:** a Department of Chemistry, Faculty of Science, Prince of Songkla University, Hat-Yai, Songkhla 90112, Thailand; b Department of Chemistry, Faculty of Science, Thaksin University, Phapayom, Phatthalung 93110, Thailand; c Faculty of Science and Technology, Hatyai University, Hat-Yai, Songkhla 90110, Thailand; d Department of Pharmaceutical Chemistry, College of Pharmacy, King Saud University, Riyadh 11451, Kingdom of Saudi Arabia; e X-ray Crystallography Unit, School of Physics, Universiti Sains Malaysia, 11800 USM, Penang, Malaysia

**Keywords:** crystal structure, aza­stilbene, anti­bacterial, anti-oxidant, hydrogen bonding

## Abstract

The title aza­stilbene derivative, C_14_H_13_NO_2_ {systematic name: (*E*)-2-[(4-meth­oxy­benzyl­idene)amino]­phenol}, is a product of the condensation reaction between 4-meth­oxy­benzaldehyde and 2-amino­phenol. The mol­ecule adopts an *E* conformation with respect to the azomethine C=N bond and is almost planar, the dihedral angle between the two substituted benzene rings being 3.29 (4)°. The meth­oxy group is coplanar with the benzene ring to which it is attached, the C_meth­yl_—O—C—C torsion angle being −1.14 (12)°. There is an intra­molecular O—H⋯N hydrogen bond generating an *S*(5) ring motif. In the crystal, mol­ecules are linked *via* C—H⋯O hydrogen bonds, forming zigzag chains along [10-1]. The chains are linked *via* C—H⋯π inter­actions, forming a three-dimensional structure.

## Abstract 2

The title compound has an *E* conformation with respect to the azomethine C=N bond and the aromatic rings are inclined to one another by 3.29 (4)°. In the crystal, mol­ecules are linked *via* C—H⋯O hydrogen bonds, forming zigzag chains along [10-1].

## Chemical context   

Aza­stilbenes have been reported to possess various biological activities such as anti­bacterial (Tamizh *et al.*, 2012 ▸), anti-oxidant (Cheng *et al.*, 2010 ▸; Lu *et al.*, 2012 ▸), anti­fungal (da Silva *et al.*, 2011 ▸) and anti­proliferative (Fujita *et al.*, 2012 ▸) including lipoxygenase inhibitor (Aslam *et al.*, 2012*b* ▸) activities. Pd^II^ and Ru^III^ complexes of aza­stilbenes have been synthesized and some have shown potent anti­bacterial activity (Briel *et al.*, 1998 ▸; Prabhakaran *et al.*, 2008 ▸; Puthilibai *et al.*, 2009 ▸). The inter­esting biological activities of aza­stilbenes have attracted us to synthesis a series of aza­stilbenes, including the title compound, and to study their anti­bacterial and anti-oxidant activities (Kaewmanee *et al.*, 2013 ▸, 2014 ▸). The anti­bacterial assay for the title compound indicated that it possesses moderate to weak anti­bacterial activity against *B. subtilis*, *S. aureus*, *P. aeruginosa*, *S. typhi* and *S. sonnei* with the MIC values in the range of 37.5 to 150 µg/ml. In addition, it also shows inter­esting anti­oxidant activity by DPPH assay with the IC_50_ value of 0.080±0.0004 µg/ml. Herein, we report on the synthesis, spectroscopic and crystallographic characterization of the title compound.
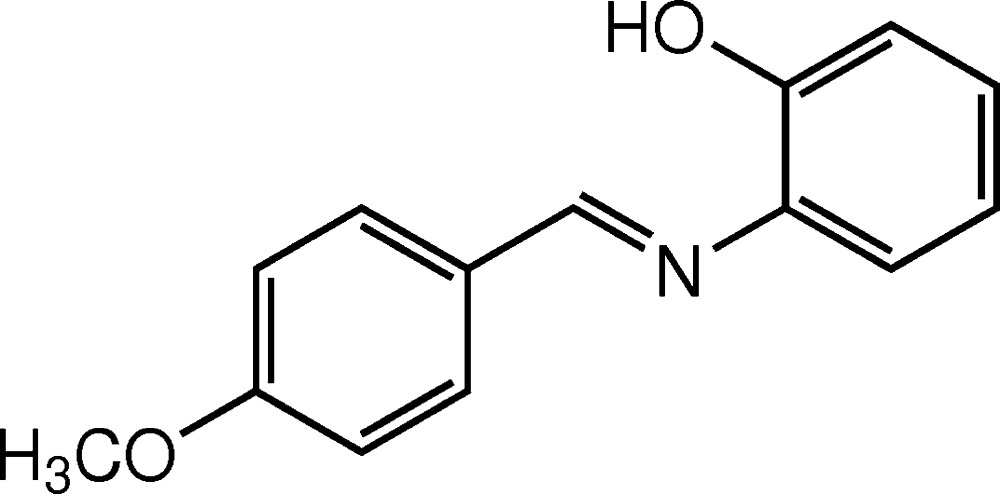


## Structural commentary   

**Figure 1:**
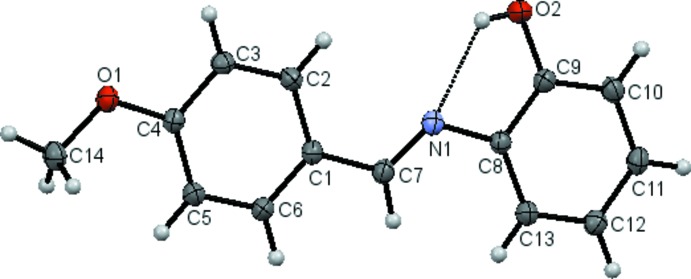
The mol­ecular structure of the title compound, with atom labelling. Displacement ellipsoids are drawn at the 60% probability level. The intramolecular O—H⋯N hydrogen bond is shown as a dashed line (see Table 1 ▸).

The title aza­stilbene compound (Fig. 1 ▸) has an *E* conformation about the azomethine C7=N1 double bond [1.2825 (10) Å], the C8—N1—C7—C1 torsion angle being −178.67 (8)°. The mol­ecule is almost planar with a dihedral angle of 3.29 (4)° between the two substituted benzene rings. The meth­oxy group is co-planar with the benzene ring to which it is attached, the C14—O1—C4—C5 torsion angle being −1.14 (12)°. There is an intra­molecular O—H⋯N hydrogen bond (Fig. 1 ▸ and Table 1 ▸) that generates an *S*(5) ring motif. The bond lengths are comparable with those found for some closely related structures (Habibi *et al.*, 2013 ▸; Aslam *et al.*, 2012*a* ▸; Kaewmanee *et al.*, 2013 ▸, 2014 ▸; Sun *et al.*, 2011 ▸).

## Supra­molecular features   

**Figure 2:**
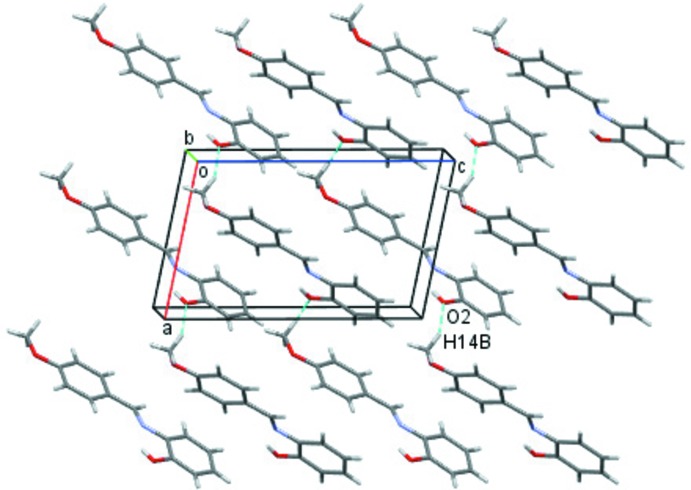
A view along the *b* axis of the crystal packing of the title compound. The C—H⋯O hydrogen bonds are shown as dashed lines (see Table 1 ▸ for details).

**Figure 3:**
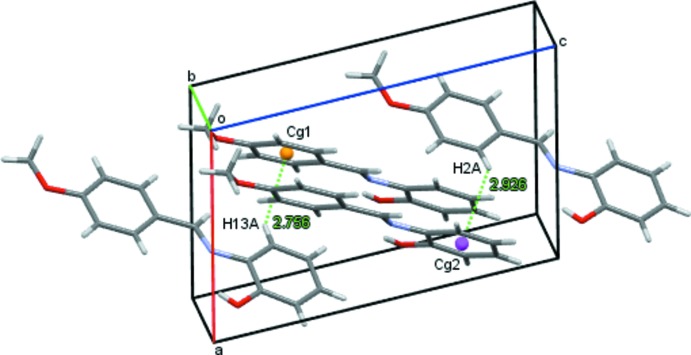
A view of the C—H⋯π inter­actions (dashed lines) in the crystal of the title compound (see Table 1 ▸ for details; ring centroids are shown as coloured spheres).

In the crystal, mol­ecules are linked *via* C—H⋯O hydrogen bonds, forming zigzag chains along [10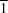] (Fig. 2 ▸ and Table 1 ▸). The chains are linked *via* C—H⋯π inter­actions (Fig. 3 ▸ and Table 1 ▸), forming a three-dimensional structure.

## Database survey   

A search of the Cambridge Structural Database (CSD, Version 5.36; Groom & Allen, 2014 ▸) for aza­stilbenes gave over 2800 hits. A search for 2-(benzyl­idene­amino)­phenols gave 78 hits, and for 2-[(4-meth­oxy­benzyl­idene)amino]­phenols there were five hits. In the compound that most closely resembles the title compound, namely 5-{[(2-hy­droxy­phen­yl)imino]­meth­yl}-2-meth­oxy­phenol (Habibi *et al.*, 2013 ▸), the two aromatic rings are inclined to one another by *ca* 16.9°.

## Synthesis and crystallization   

A solution of 4-meth­oxy­benzaldehyde (2.5 mmol, 0.37 g) in water (20 ml) and 2-amino­phenol (2.5 mmol, 0.25 g) in water (20 ml) were mixed and stirred at room temperature for around 8 h until a white precipitate appeared. The resulting white solid was filtered, washed several times with cold ethanol and then dried *in vacuo* overnight to yield the desired aza­stilbene (430 mg, 76% yield). Colourless block-shaped crystals, suitable for X-ray structure analysis, were obtained by recrystallization from methanol by slow evaporation at room temperature after several days (m.p. 388–390 K).

UV–Vis (CH_3_OH) λ_max_ (log∊): 275 (1.93), 340 (0.61) nm; FT–IR (KBr) ν: 3337, 1595, 1510, 1248, 1027 cm^−1^.; ^1^H NMR (300 MHz, DMSO-*d*_6_) δ, p.p.m.: 8.87 (*s*, 1H), 8.61 (*s*, 1H), 7.98 (*d*, *J* = 8.7 Hz, 2H), 7.18 (*dd*, *J* = 7.5, 1.2 Hz, 1H), 7.06 (*d*, *J* = 8.7 Hz, 2H), 7.03 (*td*, *J* = 7.5, 1.2 Hz, 1H), 6.83 (*td*, *J* = 7.5, 1.2 Hz, 1H), 6.09 (*dd*, *J* = 7.5, 1.2 Hz, 1H), 3.84 (*s*, –OCH_3_). The UV–Vis spectroscopic data showed absorption bands of an aza­stilbene (275 and 340 nm) while the FT–IR spectrum exhibited the stretching vibrations of O—H (3337 cm^−1^), C=N (1595 cm^−1^), C=C (1510 cm^−1^), C—N (1248 cm^−^1) and C—O (1027 cm^−1^). The successful synthesis was also supported by the ^1^H NMR spectroscopic data, which showed the characteristic signals of an olefinic proton at 8.61 (*s*, 1H) and *para*-substituted aromatic protons at 7.98 (*d*, *J* = 8.7 Hz, 2H) and 7.06 (*d*, *J* = 8.7 Hz, 2H), respectively. Moreover the ^1^H NMR spectrum also showed typical signals of *ortho*-substituted aromatic protons at 7.18 (*dd*, *J* = 7.5, 1.2 Hz, 1H), 7.03 (*td*, *J* = 7.5, 1.2 Hz, 1H), 6.83 (*td*, *J* = 7.5, 1.2 Hz, 1H) and 6.09 (*dd*, *J* = 7.5, 1.2 Hz, 1H) and a meth­oxy proton at 3.84 (*s*, –OCH_3_).

The anti­bacterial activity investigation of the title compound against Gram-positive bacteria, which are *B. subtilis*, *S. aureus*, MRSA and *E. faecalis*, and Gram-negative bacteria, which are *P. aeruginosa*, *S. sonnei* and *S. typhi*, showed moderate, mild or no inhibition. The most inter­esting anti­bacterial activity showed moderate activity against *P. aeruginosa* with an MIC value of 37.5 µg/ml.

## Refinement   

Crystal data, data collection and structure refinement details are summarized in Table 2 ▸. The OH H atom was located in a difference Fourier map and freely refined. The C-bound H atoms were positioned geometrically and allowed to ride on their parent atoms: C—H = 0.93–0.96 Å with *U*_iso_(H) = 1.5*U*_eq_(C) for methyl H atoms and 1.2*U*_eq_(C) for other H atoms.

## Supplementary Material

Crystal structure: contains datablock(s) global, I. DOI: 10.1107/S2056989015008348/su5124sup1.cif

Structure factors: contains datablock(s) I. DOI: 10.1107/S2056989015008348/su5124Isup2.hkl

Supporting information file. DOI: 10.1107/S2056989015008348/su5124Isup3.cml

CCDC reference: 1062128

Additional supporting information:  crystallographic information; 3D view; checkCIF report

## Figures and Tables

**Table 1:** Hydrogen-bond geometry (, ) *Cg*1 and *Cg*2 are the centroids of rings C1C6 and C8C13, respectively.

*D*H*A*	*D*H	H*A*	*D* *A*	*D*H*A*
O2H1*O*2N1	0.774(18)	2.078(17)	2.6315(11)	128.7(17)
C14H14*B*O2^i^	0.96	2.71	3.2876(12)	119
C2H2*A* *Cg*2^ii^	0.93	2.93	3.5662(9)	127
C13H13*A* *Cg*1^iii^	0.93	2.76	3.4671(9)	134

Symmetry codes: (i) 
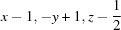
; (ii) 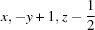; (iii) 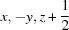.

**Table 2:** Experimental details

Crystal data	
Chemical formula	C_14_H_13_NO_2_
*M*_r_	227.25
Crystal system, space group	Monoclinic, *P* *c*
Temperature (K)	100
*a*, *b*, *c* ()	8.0357(3), 5.5554(2), 12.8733(5)
()	101.312(1)
*V* (^3^)	563.52(4)
*Z*	2
Radiation type	Mo *K*
(mm^1^)	0.09
Crystal size (mm)	0.55 0.48 0.41

Data collection	
Diffractometer	Bruker APEXII D8 Venture
Absorption correction	Multi-scan (*SADABS*; Bruker, 2009 ▸)
*T*_min_, *T*_max_	0.953, 0.964
No. of measured, independent and observed [*I* > 2(*I*)] reflections	26314, 3449, 3414
*R*_int_	0.023
(sin /)_max_ (^1^)	0.715

Refinement	
*R*[*F* ^2^ > 2(*F* ^2^)], *wR*(*F* ^2^), *S*	0.037, 0.100, 1.09
No. of reflections	3449
No. of parameters	160
No. of restraints	2
H-atom treatment	H atoms treated by a mixture of independent and constrained refinement
_max_, _min_ (e ^3^)	0.37, 0.28

Computer programs: *APEX2* and *SAINT* (Bruker, 2009 ▸), *SHELXS97*, *SHELXL97* and *SHELXTL* (Sheldrick, 2008 ▸), *Mercury* (Macrae *et al.*, 2008 ▸), *PLATON* (Spek, 2009 ▸) and *publCIF* (Westrip, 2010 ▸).

## supplementary crystallographic information

### Crystal data

**Table:**

C_14_H_13_NO_2_	*D*_x_ = 1.339 Mg m^−^^3^
*M**_r_* = 227.25	Melting point = 388–390 K
Monoclinic, *P**c*	Mo *K*α radiation, λ = 0.71073 Å
*a* = 8.0357 (3) Å	Cell parameters from 3449 reflections
*b* = 5.5554 (2) Å	θ = 2.6–30.5°
*c* = 12.8733 (5) Å	µ = 0.09 mm^−^^1^
β = 101.312 (1)°	*T* = 100 K
*V* = 563.52 (4) Å^3^	Block, colorless
*Z* = 2	0.55 × 0.48 × 0.41 mm
*F*(000) = 240	

### Data collection

**Table:**

Bruker APEXII D8 Venture diffractometer	3414 reflections with *I* > 2σ(*I*)
φ and ω scans	*R*_int_ = 0.023
Absorption correction: multi-scan (*SADABS*; Bruker, 2009)	θ_max_ = 30.5°, θ_min_ = 2.6°
*T*_min_ = 0.953, *T*_max_ = 0.964	*h* = −11→11
26314 measured reflections	*k* = −7→7
3449 independent reflections	*l* = −18→18

### Refinement

**Table:**

Refinement on *F*^2^	Secondary atom site location: difference Fourier map
Least-squares matrix: full	Hydrogen site location: inferred from neighbouring sites
*R*[*F*^2^ > 2σ(*F*^2^)] = 0.037	H atoms treated by a mixture of independent and constrained refinement
*wR*(*F*^2^) = 0.100	*w* = 1/[σ^2^(*F*_o_^2^) + (0.0786*P*)^2^ + 0.0298*P*] where *P* = (*F*_o_^2^ + 2*F*_c_^2^)/3
*S* = 1.09	(Δ/σ)_max_ < 0.001
3449 reflections	Δρ_max_ = 0.37 e Å^−^^3^
160 parameters	Δρ_min_ = −0.28 e Å^−^^3^
2 restraints	Extinction correction: *SHELXL97* (Sheldrick, 2008), Fc^*^=kFc[1+0.001xFc^2^λ^3^/sin(2θ)]^-1/4^
Primary atom site location: structure-invariant direct methods	Extinction coefficient: 0.054 (8)

### Special details

**Table:**

Experimental. The data was collected with the Oxford Cyrosystem Cobra low-temperature attachment.
Geometry. All esds (except the esd in the dihedral angle between two l.s. planes) are estimated using the full covariance matrix. The cell esds are taken into account individually in the estimation of esds in distances, angles and torsion angles; correlations between esds in cell parameters are only used when they are defined by crystal symmetry. An approximate (isotropic) treatment of cell esds is used for estimating esds involving l.s. planes.
Refinement. Refinement of F^2^ against ALL reflections. The weighted R-factor wR and goodness of fit S are based on F^2^, conventional R-factors R are based on F, with F set to zero for negative F^2^. The threshold expression of F^2^ > 2sigma(F^2^) is used only for calculating R-factors(gt) etc. and is not relevant to the choice of reflections for refinement. R-factors based on F^2^ are statistically about twice as large as those based on F, and R- factors based on ALL data will be even larger.

### Fractional atomic coordinates and isotropic or equivalent isotropic displacement parameters (Å^2^)

**Table:**

	*x*	*y*	*z*	*U*_iso_*/*U*_eq_	
O1	0.29289 (8)	0.08802 (12)	0.07528 (5)	0.01815 (14)	
O2	0.92146 (10)	0.70962 (12)	0.58775 (6)	0.02323 (15)	
H1O2	0.859 (2)	0.653 (3)	0.5407 (15)	0.028 (3)*	
N1	0.72131 (9)	0.33548 (14)	0.53665 (5)	0.01630 (16)	
C1	0.54115 (10)	0.13926 (16)	0.38967 (6)	0.01455 (16)	
C2	0.55623 (10)	0.31817 (15)	0.31478 (7)	0.01598 (16)	
H2A	0.6239	0.4524	0.3353	0.019*	
C3	0.47132 (10)	0.29620 (15)	0.21094 (7)	0.01586 (16)	
H3A	0.4826	0.4149	0.1618	0.019*	
C4	0.36790 (10)	0.09459 (15)	0.17945 (6)	0.01416 (16)	
C5	0.35026 (11)	−0.08418 (16)	0.25290 (6)	0.01607 (17)	
H5A	0.2812	−0.2171	0.2326	0.019*	
C6	0.43785 (11)	−0.05985 (16)	0.35704 (6)	0.01649 (16)	
H6A	0.4273	−0.1791	0.4061	0.020*	
C7	0.63122 (10)	0.15284 (17)	0.49969 (6)	0.01611 (17)	
H7A	0.6229	0.0242	0.5446	0.019*	
C8	0.81013 (9)	0.34278 (15)	0.64262 (6)	0.01427 (16)	
C9	0.91560 (10)	0.54613 (15)	0.66557 (6)	0.01633 (16)	
C10	1.01507 (11)	0.57944 (17)	0.76609 (7)	0.01860 (17)	
H10A	1.0854	0.7133	0.7803	0.022*	
C11	1.00837 (11)	0.41075 (16)	0.84506 (7)	0.01799 (17)	
H11A	1.0752	0.4309	0.9122	0.022*	
C12	0.90092 (10)	0.21016 (17)	0.82364 (6)	0.01711 (16)	
H12A	0.8951	0.0994	0.8770	0.021*	
C13	0.80332 (10)	0.17641 (15)	0.72308 (6)	0.01595 (16)	
H13A	0.7331	0.0424	0.7092	0.019*	
C14	0.19195 (12)	−0.11830 (17)	0.03898 (7)	0.02058 (18)	
H14A	0.1551	−0.1096	−0.0365	0.031*	
H14B	0.0948	−0.1225	0.0720	0.031*	
H14C	0.2582	−0.2615	0.0570	0.031*	

### Atomic displacement parameters (Å^2^)

**Table:**

	*U*^11^	*U*^22^	*U*^33^	*U*^12^	*U*^13^	*U*^23^
O1	0.0188 (3)	0.0212 (3)	0.0130 (3)	−0.0025 (2)	−0.0003 (2)	−0.0004 (2)
O2	0.0342 (3)	0.0193 (3)	0.0161 (3)	−0.0091 (3)	0.0045 (2)	0.0009 (2)
N1	0.0173 (3)	0.0180 (4)	0.0132 (3)	−0.0009 (2)	0.0019 (3)	−0.0010 (2)
C1	0.0157 (4)	0.0152 (3)	0.0125 (3)	−0.0003 (3)	0.0023 (3)	−0.0016 (3)
C2	0.0172 (4)	0.0142 (3)	0.0162 (4)	−0.0019 (3)	0.0024 (3)	−0.0010 (3)
C3	0.0170 (3)	0.0146 (3)	0.0155 (3)	−0.0005 (3)	0.0021 (3)	0.0013 (3)
C4	0.0133 (3)	0.0159 (4)	0.0132 (4)	0.0003 (3)	0.0026 (3)	−0.0010 (2)
C5	0.0177 (3)	0.0163 (4)	0.0142 (4)	−0.0031 (3)	0.0030 (3)	−0.0013 (3)
C6	0.0208 (4)	0.0158 (3)	0.0131 (3)	−0.0027 (3)	0.0039 (3)	−0.0002 (3)
C7	0.0176 (4)	0.0178 (4)	0.0131 (3)	−0.0008 (3)	0.0032 (3)	−0.0013 (3)
C8	0.0148 (3)	0.0150 (3)	0.0131 (3)	−0.0002 (3)	0.0029 (3)	−0.0014 (3)
C9	0.0183 (4)	0.0166 (3)	0.0148 (3)	−0.0022 (3)	0.0050 (3)	−0.0013 (3)
C10	0.0188 (4)	0.0196 (4)	0.0174 (3)	−0.0040 (3)	0.0036 (3)	−0.0042 (3)
C11	0.0172 (3)	0.0220 (4)	0.0141 (3)	−0.0008 (3)	0.0013 (3)	−0.0030 (3)
C12	0.0179 (4)	0.0184 (4)	0.0147 (4)	−0.0001 (3)	0.0025 (3)	0.0006 (3)
C13	0.0172 (3)	0.0162 (4)	0.0141 (4)	−0.0016 (3)	0.0023 (3)	−0.0005 (3)
C14	0.0194 (4)	0.0224 (4)	0.0178 (4)	−0.0031 (3)	−0.0015 (3)	−0.0028 (3)

### Geometric parameters (Å, º)

**Table:**

O1—C4	1.3586 (9)	C6—H6A	0.9300
O1—C14	1.4287 (10)	C7—H7A	0.9300
O2—C9	1.3599 (11)	C8—C13	1.3973 (11)
O2—H1O2	0.772 (19)	C8—C9	1.4084 (11)
N1—C7	1.2825 (10)	C9—C10	1.3935 (11)
N1—C8	1.4107 (10)	C10—C11	1.3915 (13)
C1—C6	1.3972 (11)	C10—H10A	0.9300
C1—C2	1.4065 (11)	C11—C12	1.4035 (12)
C1—C7	1.4605 (10)	C11—H11A	0.9300
C2—C3	1.3818 (11)	C12—C13	1.3884 (11)
C2—H2A	0.9300	C12—H12A	0.9300
C3—C4	1.4062 (11)	C13—H13A	0.9300
C3—H3A	0.9300	C14—H14A	0.9600
C4—C5	1.3975 (11)	C14—H14B	0.9600
C5—C6	1.3932 (11)	C14—H14C	0.9600
C5—H5A	0.9300		
			
C4—O1—C14	117.25 (7)	C13—C8—C9	118.98 (7)
C9—O2—H1O2	101.3 (12)	C13—C8—N1	127.73 (7)
C7—N1—C8	121.55 (7)	C9—C8—N1	113.29 (7)
C6—C1—C2	118.64 (7)	O2—C9—C10	119.93 (8)
C6—C1—C7	118.99 (7)	O2—C9—C8	119.18 (7)
C2—C1—C7	122.36 (7)	C10—C9—C8	120.88 (7)
C3—C2—C1	120.54 (7)	C11—C10—C9	119.45 (8)
C3—C2—H2A	119.7	C11—C10—H10A	120.3
C1—C2—H2A	119.7	C9—C10—H10A	120.3
C2—C3—C4	120.07 (8)	C10—C11—C12	120.11 (8)
C2—C3—H3A	120.0	C10—C11—H11A	119.9
C4—C3—H3A	120.0	C12—C11—H11A	119.9
O1—C4—C5	124.37 (7)	C13—C12—C11	120.24 (8)
O1—C4—C3	115.38 (7)	C13—C12—H12A	119.9
C5—C4—C3	120.24 (7)	C11—C12—H12A	119.9
C6—C5—C4	118.88 (7)	C12—C13—C8	120.31 (8)
C6—C5—H5A	120.6	C12—C13—H13A	119.8
C4—C5—H5A	120.6	C8—C13—H13A	119.8
C5—C6—C1	121.63 (8)	O1—C14—H14A	109.5
C5—C6—H6A	119.2	O1—C14—H14B	109.5
C1—C6—H6A	119.2	H14A—C14—H14B	109.5
N1—C7—C1	122.48 (7)	O1—C14—H14C	109.5
N1—C7—H7A	118.8	H14A—C14—H14C	109.5
C1—C7—H7A	118.8	H14B—C14—H14C	109.5
			
C6—C1—C2—C3	−0.41 (12)	C2—C1—C7—N1	4.45 (12)
C7—C1—C2—C3	178.77 (7)	C7—N1—C8—C13	−6.46 (13)
C1—C2—C3—C4	0.40 (12)	C7—N1—C8—C9	173.81 (7)
C14—O1—C4—C5	−1.14 (12)	C13—C8—C9—O2	−179.42 (8)
C14—O1—C4—C3	177.64 (7)	N1—C8—C9—O2	0.34 (11)
C2—C3—C4—O1	−178.73 (7)	C13—C8—C9—C10	1.49 (12)
C2—C3—C4—C5	0.10 (12)	N1—C8—C9—C10	−178.75 (7)
O1—C4—C5—C6	178.14 (7)	O2—C9—C10—C11	−179.85 (8)
C3—C4—C5—C6	−0.58 (12)	C8—C9—C10—C11	−0.77 (13)
C4—C5—C6—C1	0.57 (13)	C9—C10—C11—C12	−0.61 (14)
C2—C1—C6—C5	−0.09 (13)	C10—C11—C12—C13	1.27 (13)
C7—C1—C6—C5	−179.29 (7)	C11—C12—C13—C8	−0.54 (13)
C8—N1—C7—C1	−178.67 (8)	C9—C8—C13—C12	−0.82 (12)
C6—C1—C7—N1	−176.38 (8)	N1—C8—C13—C12	179.45 (7)

### Hydrogen-bond geometry (Å, º)

Cg1 and Cg2 are the centroids of rings C1–C6 and C8–C13, respectively.

**Table:**

*D*—H···*A*	*D*—H	H···*A*	*D*···*A*	*D*—H···*A*
O2—H1*O*2···N1	0.774 (18)	2.078 (17)	2.6315 (11)	128.7 (17)
C14—H14*B*···O2^i^	0.96	2.71	3.2876 (12)	119
C2—H2*A*···*Cg*2^ii^	0.93	2.93	3.5662 (9)	127
C13—H13*A*···*Cg*1^iii^	0.93	2.76	3.4671 (9)	134

Symmetry codes: (i) *x*−1, −*y*+1, *z*−1/2; (ii) *x*, −*y*+1, *z*−1/2; (iii) *x*, −*y*, *z*+1/2.
